# Developing a community-based genetic nomenclature for anole lizards

**DOI:** 10.1186/1471-2164-12-554

**Published:** 2011-11-11

**Authors:** Kenro Kusumi, Rob J Kulathinal, Arhat Abzhanov, Stephane Boissinot, Nicholas G Crawford, Brant C Faircloth, Travis C Glenn, Daniel E Janes, Jonathan B Losos, Douglas B Menke, Steven Poe, Thomas J Sanger, Christopher J Schneider, Jessica Stapley, Juli Wade, Jeanne Wilson-Rawls

**Affiliations:** 1 School of Life Sciences, Arizona State University, PO Box 874501, Tempe, AZ 85287-4501, USA; 2 Department of Biology, Temple University, 1900 N. 12th Street, Philadelphia, PA 19122, USA; 3 Department of Organismic and Evolutionary Biology, Harvard University, 16 Divinity Ave., Cambridge, MA 02138, USA; 4 Department of Biology, Queens College, The City University of New York, 65-30 Kissena Boulevard, Flushing, NY 11367-1597; USA; 5 Department of Biology, Boston University, 5 Cummington Street, Boston, MA 02215, USA; 6 Department of Ecology and Evolutionary Biology, University of California, 621 Charles E. Young Drive South, Los Angeles, CA 90095, USA; 7 Department of Environmental Health Science, University of Georgia, 150 East Green Street, Athens, GA 30602, USA; 8 Museum of Comparative Zoology, Harvard University, 26 Oxford St., Cambridge, MA 02138, USA; 9 Department of Genetics, University of Georgia, 120 East Green Street, Athens, GA 30602-7223, USA; 10 Department of Biology, University of New Mexico, 167 Castetter Hall, MSC03 2020, 1 University of New Mexico, Albuquerque, NM 87131-0001, USA; 11 Smithsonian Tropical Research Institute, Unit 9100 BOX 0948, DPO AA 34002-9998, USA; 12 Departments of Psychology and Zoology, Michigan State University, 212 Giltner Hall, East Lansing, MI 48824-1101, USA

## Abstract

**Background:**

Comparative studies of amniotes have been hindered by a dearth of reptilian molecular sequences. With the genomic assembly of the green anole, *Anolis carolinensis *available, non-avian reptilian genes can now be compared to mammalian, avian, and amphibian homologs. Furthermore, with more than 350 extant species in the genus *Anolis*, anoles are an unparalleled example of tetrapod genetic diversity and divergence. As an important ecological, genetic and now genomic reference, it is imperative to develop a standardized *Anolis *gene nomenclature alongside associated vocabularies and other useful metrics.

**Results:**

Here we report the formation of the *Anolis *Gene Nomenclature Committee (AGNC) and propose a standardized evolutionary characterization code that will help researchers to define gene orthology and paralogy with tetrapod homologs, provide a system for naming novel genes in *Anolis *and other reptiles, furnish abbreviations to facilitate comparative studies among the *Anolis *species and related iguanid squamates, and classify the geographical origins of *Anolis *subpopulations.

**Conclusions:**

This report has been generated in close consultation with members of the *Anolis *and genomic research communities, and using public database resources including NCBI and Ensembl. Updates will continue to be regularly posted to new research community websites such as *lizardbase*. We anticipate that this standardized gene nomenclature will facilitate the accessibility of reptilian sequences for comparative studies among tetrapods and will further serve as a template for other communities in their sequencing and annotation initiatives.

## Background

As the rate of generating new sequence assemblies continues to accelerate, the final bottleneck that remains is annotation. While automated pipelines have been developed, it is still up to community initiatives to pool, evaluate, integrate, and disseminate the necessary resources required for functional and comparative annotations that support research needs. The presence of multiple tools and resources, and changing assemblies and annotations, presents "moving-target" challenges for those attempting to assign function, orthology, nomenclature and other common vocabulary to genetic loci. One challenge is that many assemblies are, or will be, periodically updated due to resequencing efforts that aim to fill in ever-present gaps, initiatives to provide a consensus reference sequence that takes into account the polymorphism present in a species, or a re-deployment of different assembly algorithms. The second challenge is that the generation of confidently assigned gene models on a fixed assembly generally correlates with the amount of effort that a community puts into annotating their genome of interest. A third challenge relates to the principle that orthologous (and by association, functional) assignments are interdependent on the quality and quantity of annotations from closely related genomes.

The recent publication of the genome sequence of the green anole, *Anolis carolinensis*, offers a rich trove of opportunities for biologists [1]. Comparing vertebrate genomes holds the promise to solve such questions as unmasking the genetic basis of human disease in addition to understanding common evolutionary processes. Whole genome sequencing efforts in vertebrates have been carried out for 39 species of mammals (10 primates, 8 rodents, 12 laurasiatherians, 3 afrotherians, 2 xenarthrans, 3 marsupials, 1 monotreme), 3 birds (avian reptiles), 1 amphibian, and 5 teleost species [2,3]. Non-avian reptiles are missing from this taxonomic survey of genomes, and the publication of a whole genome assembly for the green anole helps to fill this gap [1]. As a complement to this effort, a growing number of online resources are available for the *Anolis *community (Table 1).

**Table 1:** *Anolis *online databases and resources

db Name	Resources/Tools Available	URL
*Anole Annals*	• Blog updated regularly and focused on the latest *Anolis *research	http://www.anoleannals.wordpress.com
*Anolis Genome*	• *Anolis *genomic and expression data	http://www.anolisgenome.org
*Anolis Genome Project*	• Primary site for genome sequencing effort by the Broad Institute	http://www.broadinstitute.org/models/anole
*Anolis Newsletter*	• Manuscripts and reports generated by the *Anolis *community	http://anolis.oeb.harvard.edu
*Ensembl*	• *Anolis carolinensis *portal, genome and annotations	http://www.ensembl.org/Anolis_carolinensis/Info/Index
*lizardbase*	• *Anolis *genome browser **• **GIS data mapping • Gene nomenclature resources • *Anolis *educational materials	http://www.lizardbase.org
*NCBI Unigene*	• *Anolis carolinensis *transcripts	http://www.ncbi.nlm.nih.gov/UniGene/UGOrg.cgi?TAXID=28377
*UCSC*	• *Anolis carolinensis *portal • Comparative genomic tracks	http://www.genome.ucsc.edu/cgi-bin/hgGateway

Mammals, birds, and non-avian reptiles are grouped as amniotes, due to shared features including a characteristic egg adapted to terrestrial reproduction. Within the amniotes, mammals are estimated to have diverged over 300 million years ago (mya) from the reptiles [4]. Within the Reptilia are three major lineages: the Archosauria, which contains crocodilians, dinosaurs and birds and whose most recent common ancestor lived approximately 250 mya; the Lepidosauria, which contains the Squamata (lizards and snakes) and the tuatara (a lizard-like reptile found only in New Zealand); and the Anapsida or turtles. For comparative genomic analysis, this first non-avian reptile sequence will be invaluable as an outgroup for comparative analyses of an increasing number of amniote sequences.

For the past century, *A. carolinensis*, which is native to the southeastern US, has been a lizard of choice for comparative studies in ecology, evolutionary biology, behavior, physiology and neuroscience. With genomic and transcriptomic sequences available, *A. carolinensis *is also emerging as an important model organism for cellular, molecular, developmental and regenerative studies. Furthermore, *A. carolinensis *is only one of over 350 described species of *Anolis*, making it a member of one of the most species-rich clades of tetrapods [4].

Comparative genomic research at all taxonomic levels would be facilitated by a consistent system of gene nomenclature for *A. carolinensis *as the first sequenced non-avian reptile. Towards this goal, members of the *Anolis *research community have established the *Anolis *Gene Nomenclature Committee (AGNC) to generate and maintain standardized gene vocabularies. As a companion to the publication of the first non-avian reptile genome, we present this report as the first step in an evolving document.

## Report and Discussion

### Establishing evolutionary metrics to help evaluate orthology between anoles and other vertebrates

As an approach in the annotation process, finding orthologous relationships across species has become an important tool to evaluate gene identity [5]. However, determining gene orthology is not a trivial exercise. Vertebrate genomes have experienced a dynamic flux of activity from countless deletions and duplications, a constant stream of genomic rearrangements (including at least two whole genome duplications), and divergence in both gene expression and protein function. Fortunately, for many genes, orthologs can be reliably determined based on reciprocal protein similarity. For other genes, divergence in sequence requires data from synteny (gene order) conservation and functional analysis to also be considered. Below, we present the challenges involved in maintaining an evolving and community-accepted record of gene ancestry, and briefly review the current state of assigning orthology using presently available resources and tools. Proposed criteria for evaluating gene orthology and paralogy are offered below with an aim to present a multi-metric summary for each gene that offers a measure of the confidence with which the investigator can assign orthology.

#### Resources and challenges for assigning orthology

##### Confidence in genome assembly

High quality whole genome assemblies are essential for confidence in comparative analysis. The genome of *A. carolinensis *(estimated to be 1.78 Gbp) was first assembled in March 2007 via shotgun reads to a depth of 6.85X (AnoCar1.0) [1]. The second iteration of genome assembly (AnoCar2.0) was released in May 2010 and included increased coverage (7.10X). The Anocar2.0 assembly incorporated 6,645 scaffolds comprised of 41,985 contigs with a supercontig N50 of 4.0 Mbp. Scaffolds were anchored to chromosomes by FISH mapping using 405 BACs. Increased genome coverage from new sequencing efforts is anticipated in the upcoming years. Improved assemblies will allow for conserved syntenic blocks to be more easily recognized thereby greatly assisting in identifying orthologs with confidence.

##### Confidence in gene models

Our inference of gene orthology depends on the quality of gene annotations among the multiple species compared. Awaiting large public genome databases such as EMBL-EBI/Sanger's Ensembl and NCBI's UniGene to generate gene models and clusters provides a trouble-free route to reliable annotations; however, the lag time from assembly release to initiating an annotation build currently remains at least four months and can take over an entire year to become publicly available. Presently, Ensembl generates a fairly quick and reliable gene build that is based on a combination of *ab initio *gene predictions, comparative genomics, and incorporation of experimental (e.g., ESTs) resources (doi:10.1101/gr.1858004). Ensembl GeneBuild58.1b dramatically increased the number of genes annotated in *A. carolinensis *from a pre-genome list of 36 loci to a genome-wide set (based on AnoCar1.0) of 11,932 loci. Of these initial annotations, 4,793 new genes were discovered along with 471 pseudogenes and 3,099 RNA genes comprising a total count of 20,885 transcripts. In contrast, UniGene clusters ESTs and mRNAs: as a result UniGene Build version 2 described 26,575 transcript clusters. So, how do we compare the quality of each of these annotation sets? An interesting feature used by some model organism databases is the application of confidence scores. In FlyBase [6] a single digit scoring metric is assigned based on evaluating three different classes of evidence: *ab initio *gene prediction algorithms, aligned nucleotide sequences and overlapping regions of protein similarity. FlyBase plans to refine their transcript confidence to include support from comparative genomics, proteomic analyses, and to potentially provide details on the magnitude and quality of each type of support. Comparable approaches are planned to be developed for *A. carolinensis *(see below).

##### Confidence in aligned assemblies from nearby taxa

The paucity of amphibian and reptilian sequences compared with mammalian genomes presents a challenge for comparative analysis. When entire vertebrate clades depend on the annotations of a single genome, errors in comparative analysis are likely. As more annotated assemblies become available, we should be able to test and refine current assignments of orthologous and paralogous relationships. Yet, not all annotations are created equally, with model organisms such as chicken, mouse, rat and zebrafish having more comprehensive annotations due to greater allocated resources and larger active research communities. Therefore, the challenge is to develop an annotation approach that keeps pace with the rapidly expanding number of whole genome sequences being produced.

##### Currently available orthology pipelines

Ancestral relationships between loci from selected species can be extracted via a variety of ready-built pipelines. The major databanks provide orthology/paralogy relationships for completed genomes through the implementation of well-established data workflows. Ensembl's orthology and paralogy relationships are based on a maximum likelihood tree-building algorithm, TreeBeST [7]. NCBI's Homologene uses a clustering approach based on an initial blastp search [8]. The UCSC Genome Browser also generates a comparative genomic table on selected sequenced species [9,10]. A number of other databases that specifically identify orthology/homology include the Orthologs Matrix Project (OMA) [11,12], InParanoid [13,14], TreeFam [15,16], Optic [17,18], and Evola [19,20]. Interestingly, HUGO (Human Genome Organization) has constructed a meta-comparison tool, HCOP (Human Gene Nomenclature Committee Comparison of Orthology Predictions), that records whether an orthology call has evidence in each of the before-mentioned pipelines, hence, providing a valuable evaluative resource to assess overall confidence [21]. A major challenge for bioinformatics research is to keep up with an ever-changing landscape of software tools. Workflow evaluations must be performed on a regular basis by computer-savvy researchers but, most importantly, the results must be validated by knowledgeable biologists.

#### Towards community-driven evaluations of orthology

With an accelerated increase in genomic sequence data, even a well-organized mechanism to assign orthology can be overwhelmed. A community-driven effort to characterize a gene's evolutionary history as well as our confidence in summarizing it will be useful to the community and beyond. We propose that the *Anolis *research community work together to initiate and ultimately complement these efforts to build a pipeline that follows a common set of guidelines and relationships with the large genomic databanks. Towards this end, the AGNC has established working relationships with representatives from a network of relevant databases.

Developing a common set of guidelines is the major focus of the AGNC in the upcoming year. Ultimately, we aim to generate a weighted point system, considering the different types of characteristics being compared. In situations where there is still substantial ambiguity, the AGNC plans to work with the researchers and database community for preliminary assignments. In the interim, we propose the following framework as a starting point:

##### Species/taxa for comparative analysis

Multiple alignment programs such as ClustalW [22], MUSCLE [23] and T-COFFEE [24] provide accessible tools to align multiple species. The presence or absence of reliable alignments can tell us which lineage this gene is limited to. All comparative analyses should include a common starting set of genomes to align to:

• Mammals: 2 eutherians, preferably mouse and human, plus marsupial and monotreme genes if available.

• Birds (avian reptiles): zebra finch and chicken

• Non-avian reptiles: Any additional gene sequences as available, particularly for non-squamate species (turtles or crocodilians)

• Amphibians: *Xenopus tropicalis *and additional genomes as available

• Teleosts: Zebrafish and *Fugu rubripes *or *Tetraodon nigroviridis *should be included. Additional teleosts (stickleback, medaka) can also be analyzed.

• Non-vertebrate chordates: Either *Ciona intestinalis *or *savignyi *can serve as a stem alternative to *Drosophila melanogaster*, if available.

##### Protein sequence analysis

Sequence analysis programs such as MEGA [25] and PAML [26] provide accessible tools to analyze protein alignment across multiple species. Protein divergence will be estimated using dN (amino acid divergence) and dS (silent site divergence) using a codon-substitution matrix. There will be much variation in divergence estimates across proteins; however, confidence in alignment can be evaluated by comparing these estimates to other proteins. In particular, dS will serve as a neutral divergence marker among vertebrates while dN will provide a rough indicator of sequence alignment quality across larger phylogenetic distances.

##### Orthology/Paralogy relationships

Using the alignments, it will be informative to extract copy number information for each gene. A number of databases also provide this information (e.g., Ensembl) in their orthology pipelines. Relationships such as 1:1, 1:n, n:n (where n is an integer) are instructive to users interested in gene families and how they evolved between lizards and a reference genome such as chicken.

##### Predicted transcript sequence analysis

Building on an approach used by FlyBase [6], each transcript receives a score based on a single-digit octal notation and the sum of the following categories (to an 8 point maximum):

• 1 point if one or more aligned EST sequences aligns to the annotated transcript,

• 2 points if an annotated exon intersects a region of aligned protein similarity (of course, similarity to self is excluded),

• 4 points if there is any gene prediction that is fully consistent with the annotated transcript, and

• 8 points if one or more aligned cDNAs are fully consistent with the annotated transcript.

##### Experimentally defined transcript sequence and alternative splicing

EST or full-length cDNA transcript sequence is highly preferable to predicted annotations and should be used at every opportunity. Suggested parameters are currently as defined above. For alternative splicing, the identification of similar patterns of alternative splicing in the species being compared greatly increases confidence that there is an orthologous relationship.

##### Synteny conservation

Minimally, orthology could be recognized by the presence of at least 2 orthologous genes, from *Gallus gallus*, on either the 5' or 3' flanking sequences and in sequential order. Confidence increases with additional orthologous genes on one flank, or synteny conservation on both flanking regions.

##### Gene expression

Following gene duplication events, divergence of regulatory control regions can lead to differentiation in tissue specificity and timing of gene expression in paralogous genes. These regulatory regions are considered part of the gene being compared, but it is not straightforward to assign a score to this divergence. Genes that appear to be orthologous by the measures above can still display strikingly different gene expression, raising the question of whether the regulatory gene functionality has diverged in an opposing fashion to that of the protein coding sequence. This is one of the most difficult comparisons to evaluate, and as more comparative analyses are reported, the AGNC aims to develop proposals regarding how genes should be annotated when sequence and expression suggest contradictory findings about the descent of gene functionality.

Much of the above information can be collated into a single colon-separated string that provides the AGNC with a single metric to evaluate nomenclature, and the user with an instant confidence metric. Since this evolutionary character code (ECC) would change depending on the input data, the metric would simply be linked to the gene as a separate feature. As an example, a hypothetical "gene2" would be annotated with the gene description, gene2:chordates:80,55:1-1:5:3,4:TS, meaning that gene2 has orthology only within chordates with, respectively, 80% and 55% overall protein and nucleotide identity (alternatively, dN and dS can be used), it doesn't possess paralogs within and between species (chicken), it has both gene prediction and EST evidence (an octal score of 5), 3 genes upstream with synteny conservation with the reference species and 4 genes downstream, and tissue-specific expression in a cross-species comparison (e.g., with mouse).

With the adoption of a reliable set of orthologous relationships, downstream functional and comparative annotations and alignments that can be used by the entire community could quickly be generated. As an example, gene ontologies (GO) can be easily transferred after orthologies are assigned. Since the chicken genome is one of the twelve "reference" genomes that the Gene Ontology database is carefully annotating with controlled ontological vocabulary [27], the *A. carolinensis *genome is in an excellent position to be annotated reliably with associated GO terms.

These data must be quickly disseminated to the community via regularly updated databases. The *Anolis *community currently has a database that is preparing for the next generation of data sets. *lizardbase *[28] is the primary community website and anole resource that includes a mapping portal for both geographical and genome-based data. It is critical that such community-serving databases coordinate the effort to provide consensus datasets.

### Nomenclature for *Anolis *gene names and symbols

Analysis of the chicken and zebra finch genomes has demonstrated that while a majority of genes can be assigned clear orthologs, functional genes unique to the avian lineage require additional analysis [29]. With the *A. carolinensis *genome, the challenge is for gene nomenclature to both clearly point out orthology with other vertebrates and allow for identification of non-avian, reptile-specific genes. The AGNC has reviewed guidelines issued by gene nomenclature organizations from mammalian (Human Gene Nomenclature Committee, HUGO; International Committee on Standardized Gene Nomenclature for Mice), avian reptile (Chicken Gene Nomenclature Committee) [30], amphibian (Xenbase) [31,32], and teleost (ZFIN, Zebrafish Information Network) [33,34] communities.

A major consideration for gene nomenclature in *A. carolinensis *is flexibility for comparisons with other amniote genomes. Given that the most frequent comparisons of *Anolis *genes would likely be with human, mouse, or chicken orthologs, the AGNC proposes using a gene symbol style that would allow the reader to infer the species based on the symbol alone. For a hypothetical gene named "gene2", likely species for cross-comparison are:

*GENE2*, human (*Homo sapiens*): all capitals, italicized

*Gene2*, mouse (*Mus musculus*): first letter capitalized, italicized

*GENE2*, chicken (*Gallus gallus*): all capitals, italicized

*gene2*, *Xenopus tropicalis*: all lower case, italicized

*gene2*, zebrafish (*Danio rerio*): all lower case, italicized

To make it easier to distinguish a reference to an *Anolis *gene in comparisons with human, mouse, and avian orthologs, the AGNC proposes a gene symbol style similar to *Xenopus tropicalis *and zebrafish, i.e.,

*gene2, Anolis carolinensis*: all lower case, italicized

Further details of these guidelines are presented below.

#### Gene symbols

• Gene symbols for all *Anolis *species should be written in lower case only and in italics, e.g., *gene2*.

• Whenever criteria for orthology have been met (previous Section), the *Anolis *gene symbol should be comparable to the human gene symbol, e.g., if the human gene symbol is *GENE2*, then the *Anolis *gene symbol would be *gene2*. In situations where the human and mouse symbols differ, the AGNC requests that the investigator contact the AGNC through *lizardbase *to determine a suitable gene symbol for *Anolis*.

• Orthologous genes in other *Anolis *species should have the same gene symbol and name as *A. carolinensis*. A proposed abbreviation code system for comparisons within the genus covering *Anolis *species is presented below (see section below; Table 1).

• Gene symbols should only contain ASCII characters (Latin alphabet, Arabic numerals)

• Punctuation (dashes, periods, slashes) should not be used unless they are part of a human or mouse gene symbol, e.g., if the human gene symbol is *NKX3-1*, then the *Anolis *gene symbol should be *nkx3-1*.

• Gene names: In other model systems, a unique database of gene symbols is typically maintained by a gene nomenclature committee, but there is more variability for the full gene name. Whenever possible, the human or mouse gene name should be used, but omitting references to homology or disease descriptions, e.g., "delta-like 1", not "delta-like 1 (*Drosophila*)". Provisional human or mouse gene names, e.g., KIAA# or C#orf, should not be used as the basis for a gene name in *Anolis *species.

• Novel gene names and symbols: If an orthologous gene cannot be identified in any currently sequenced genome, a novel name may be selected by the investigators. The name should ideally be brief and convey information about the gene expression or function but not include proper or commercial names, e.g., *yep1*, yolk expressed protein 1. References to molecular weight should be avoided, i.e., do not use *p35*, 35 kDal protein.

• Gene symbols should not start with an "A" or "Ac" as an abbreviation for *Anolis carolinensis*, i.e., not *acgene2*. Gene symbols may start with "a" or "ac" if the human or mouse ortholog starts with these letters, e.g., *actb *for beta-actin.

• Using criteria for orthology described in the previous objective, duplication of the *Anolis *ortholog of a mammalian gene will be indicated by an "a" or "b" suffix, e.g., *gene2a *and *gene2b*. If the mammalian gene symbol already contains a suffix letter, then there would be a second letter added, e.g., *gene4aa *and *gene4ab*.

#### Protein symbols

• Protein symbols should be the same as the gene symbol except written in all upper case without italics, e.g., GENE2.

### Nomenclature for *Anolis *non-coding sequences, including transposons and repetitive elements

The classification and nomenclature of transposable elements presents a particular challenge because of the large diversity of transposons in eukaryotic genomes. Several classification and naming schemes have been proposed but there is currently no consensus on how transposons should be annotated [35,36]. An ideal classification system of transposable elements should reflect the evolutionary relationships among elements [37]. However, as eukaryotic genomes are annotated independently from each other there has been a tendency to name transposon families by numbering them in the order they are discovered, without much consideration of their evolutionary affinities across genomes [38]. Although scientists agree on the major categories of transposable elements (DNA transposons, non-LTR retrotransposons and LTR retrotransposons), there is no consensus on their classification at lower levels (families and subfamilies) and on how to name newly discovered transposons. Thus, the nomenclature of transposons can be considered a work in progress. An International Committee on the Classification of Transposable Elements has been created and is aiming to build a classification that will reflect the structural and evolutionary affinities among elements, yet that will also be relatively easy to use. Until a consensus is reached within the transposable element community, we propose some simple guidelines for the nomenclature of transposable elements in *A. carolinensis*.

The general principles of the nomenclature follow the recommendations of Kapitonov and Jurka [37], with some minor modifications. Kapitonov and Jurka proposed to name elements by the super-family in which they belong, followed by a unique identifier (generally a number), a structural identifier if necessary, and end with a species identifier. For example, *Helitron-1_Acar *would be the name of family 1 of autonomous *Helitron *in *A. carolinensis*. If a non-autonomous family of *helitron *has been amplified by *Helitron-1_Acar*, its name will be *Helitron-1N1_Acar*, the *N *indicating its non-autonomous nature. However, the diversity within some super-families is relatively well known, at least in vertebrates, and we propose that the name of elements should reflect their evolutionary affinities below the super-family level. For instance, the *hAT *super-family contains several well-defined monophyletic lineages (e.g., *hobo*, *Charlie*, *restless*). In those cases where the diversity of the super-family is well characterized, we propose to name elements using the name of the clades. For instance, we propose to use the name *hobo-1_Acar *instead of *hAT-1_Acar *for a family that is unambiguously related to other *hobo *elements.

An additional difficulty in naming transposable elements results from the common occurrence of horizontal transfer. A consequence of horizontal transfer is that identical or very similar elements might be found in distantly related organisms [39-42]. Novick et al. [41] proposed to use the letter *HT *to indicate the fact that an element has been horizontally transferred from another species, e.g. *hAT-HT1_Acar*. However, this solution is not satisfactory as the same elements might carry different names in different organisms because genomes are annotated independently. For instance, the anole *hAT-HT2_Acar *is different from the *hAT2_ML *of bats but is identical to the *hAT4 *in *Xenopus tropicalis*. In those cases, we believe it is better to not use a numbering scheme but instead to choose a different name for those families that are found in distantly related taxa. A name that reflects at least partially the evolutionary affinities of the elements is preferable. The solution adopted in Thomas et al. [42] to name horizontally transferred *helitrons *seems satisfactory, e.g., *Heligloria*.

As mentioned earlier, the classification and nomenclature of transposons is a work in progress that will require a better knowledge of transposable element evolution below the super-family level and across genomes. It is the goal of the committee to regularly improve and update the classification of *A. carolinensis *elements.

### Abbreviations for *Anolis *species and population groups

Comparative and functional genomics is rapidly progressing from broad-scale comparisons among model systems to fine-scale analyses among populations and closely related species [43-45]. *Anolis *is an ecologically, physiologically, and morphologically diverse genus of over 350 species that has a rich history of comparative studies [4]. While the nomenclature described above establishes guidelines for the model system, *A. carolinensis*, it is critical that the research community arrive at a common vocabulary to reference data from other *Anolis *species and among populations. The AGNC proposes the following guidelines with this aim:

• All genus and species abbreviations for anoles will begin with the capital letter, 'A', followed by three lowercase italicized letters based approximately on the first letters of the species name, e.g., *Anolis sagrei *= *Asag*.

• In comparative analyses abbreviations will be added as a suffix to the proper gene names, *e.g., gene2-Asag*.

• The three-letter species abbreviation suffix (in lowercase) is generated by the first two letters of the species name and an identifying third letter unique to each species. In cases of redundancy in all of the first three letters of species names, precedence is given to the date of first publication. For the remaining species, the third letter will be replaced with the subsequent letter of the species name that generates a unique code. Examples: *A. grahami *= *Agra *since this species was first reported in 1845 [46]; *A. gracilipes *= *Agrc*; *A. granuliceps *= *Agrn*. A full listing of 378 abbreviations based on our current view of the species content of *Anolis *is found in Table 2 and posted to various anole community sites listed at the end of this report.

**Table 2:** *Anolis *species and proposed abbreviations

*Anolis *species	Abbreviation
*acutus*	*Aacu*
*aeneus*	*Aaen*
*aequatorialis*	*Aaeq*
*agassizi*	*Aaga*
*agueroi*	*Aagu*
*ahli*	*Aahl*
*alayoni*	*Aala*
*alfaroi*	*Aalf*
*aliniger*	*Aali*
*allisoni*	*Aals*
*allogus*	*Aall*
*altae*	*Aalt*
*altavelensis*	*Aalv*
*altitudinalis*	*Aaln*
*alumina*	*Aalm*
*alutaceus*	*Aalu*
*alvarezdeltoroi*	*Aald*
*amplisquamosus*	*Aamp*
*anatoloros *	*Aana*
*anchicayae*	*Aanc*
*anfilioquioi*	*Aanf*
*angusticeps*	*Aang*
*anisolepis*	*Aani*
*annectens*	*Aann*
*antioquiae*	*Aano*
*antoni*	*Aant*
*apletophallus *	*Aapl*
*apollinaris*	*Aapo*
*aquaticus*	*Aaqu*
*argenteolus*	*Aarg*
*argillaceus*	*Aari*
*armouri*	*Aarm*
*auratus*	*Aaur*
*baccatus*	*Abac*
*bahorucoensis*	*Abah*
*baleatus*	*Abal*
*baracoae*	*Abao*
*barahonae*	*Aban*
*barbatus*	*Abab*
*barbouri*	*Abar*
*barkeri*	*Abak*
*bartschi*	*Abat*
*beckeri*	*Abec*
*bellipeniculus*	*Abel*
*bicaorum*	*Abic*
*bimaculatus*	*Abim*
*binotatus*	*Abin*
*biporcatus*	*Abip*
*birama*	*Abir*
*biscutiger*	*Abis*
*bitectus*	*Abit*
*blanquillanus*	*Abla*
*boettgeri*	*Aboe*
*bombiceps*	*Abom*
*bonairensis*	*Abon*
*bouvieri*	*Abou*
*breedlovei*	*Abrd*
*bremeri*	*Abrm*
*brevirostris*	*Abre*
*brunneus*	*Abru*
*calimae*	*Acal*
*campbelli*	*Acam*
*capito*	*Acap*
*caquetae*	*Acaq*
*carlostoddi*	*Acao*
*carolinensis*	*Acar*
*carpenteri*	*Acae*
*casildae*	*Acas*
*caudalis*	*Acau*
*centralis*	*Acen*
*chamaeleonides*	*Acha*
*charlesmeyeri*	*Ache*
*chloris*	*Achi*
*chlorocyanus*	*Achl*
*chocorum*	*Acho*
*christophei*	*Achs*
*chrysolepis*	*Achr*
*clivicola*	*Acli*
*cobanensis*	*Acob*
*coelestinus*	*Acoe*
*compressicauda*	*Acom*
*concolor*	*Acon*
*confusus*	*Acof*
*conspersus*	*Acos*
*cooki*	*Acoo*
*crassulus*	*Acra*
*cristatellus*	*Acri*
*cristifer*	*Acrs*
*cryptolimifrons*	*Acry*
*cumingi*	*Acum*
*cupeyalensis*	*Acue*
*cupreus*	*Acup*
*cuprinus*	*Acur*
*cuscoensis*	*Acuc*
*cusuco*	*Acus*
*cuvieri*	*Acuv*
*cyanopleurus*	*Acya*
*cybotes*	*Acyb*
*cymbops*	*Acym*
*damulus*	*Adam*
*danieli*	*Adan*
*darlingtoni*	*Adar*
*datzorum*	*Adat*
*delafuentei*	*Adef*
*deltae*	*Adel*
*desechensis*	*Ades*
*dissimilis*	*Adii*
*distichus*	*Adis*
*dolichocephalus*	*Adoi*
*dollfusianus*	*Adol*
*dominicanus*	*Adom*
*duellmani*	*Adue*
*dunni*	*Adun*
*eewi*	*Aeew*
*electrum*	*Aele*
*equestris*	*Aequ*
*ernestwilliamsi*	*Aern*
*etheridgei*	*Aeth*
*eugenegrahami*	*Aeug*
*eulaemus*	*Aeul*
*euskalerriari*	*Aeus*
*evermanni*	*Aeve*
*extremus*	*Aext*
*fairchildi*	*Afai*
*fasciatus*	*Afas*
*ferreus*	*Afer*
*festae*	*Afes*
*fitchi*	*Afit*
*forbesi*	*Afor*
*fortunensis*	*Afot*
*fowleri*	*Afow*
*fraseri*	*Afra*
*frenatus*	*Afre*
*fugitivus*	*Afug*
*fungosus*	*Afun*
*fuscoauratus*	*Afus*
*gadovi*	*Agad*
*garmani*	*Agar*
*garridoi*	*Agai*
*gemmosus*	*Ager*
*gibbiceps*	*Agib*
*gingivinus*	*Agin*
*godmani*	*Agod*
*gorgonae*	*Agor*
*gracilipes*	*Agrc*
*grahami*	*Agra*
*granuliceps*	*Agrn*
*greyi*	*Agre*
*griseus*	*Agri*
*gruuo*	*Agru*
*guafe*	*Aguf*
*guamuhaya*	*Agua*
*guazuma*	*Aguz*
*gundlachi*	*Agun*
*haetianus*	*Ahae*
*haguei*	*Ahag*
*hendersoni*	*Ahen*
*heterodermus*	*Ahet*
*heteropholidotus*	*Ahee*
*hobartsmithi*	*Ahob*
*homolechis*	*Ahom*
*huilae*	*Ahui*
*humilis*	*Ahum*
*ibague*	*Aiba*
*ibanezi*	*Aibn*
*imias*	*Aimi*
*impetigosus*	*Aimp*
*incredulus*	*Ainc*
*inderenae*	*Aind*
*inexpectata*	*Aine*
*insignis*	*Ains*
*insolitus*	*Aino*
*isolepis*	*Aiso*
*isthmicus*	*Aist*
*jacare*	*Ajac*
*johnmeyeri*	*Ajoh*
*juangundlachi*	*Ajua*
*jubar*	*Ajub*
*kemptoni*	*Akem*
*koopmani*	*Akoo*
*kreutzi*	*Akre*
*krugi*	*Akru*
*kunayalae*	*Akun*
*laevis*	*Alav*
*laeviventris*	*Alae*
*lamari*	*Alam*
*latifrons*	*Alat*
*leachi*	*Alea*
*lemniscatus*	*Alen*
*lemurinus*	*Alem*
*limifrons*	*Alim*
*lineatopus*	*Alie*
*lineatus*	*Alin*
*liogaster*	*Alig*
*lionotus*	*Alio*
*litoralis*	*Alit*
*lividus*	*Aliv*
*longiceps*	*Alon*
*longitibialis*	*Alog*
*loveridgei*	*Alov*
*loysianus*	*Aloy*
*luciae*	*Alua*
*lucius*	*Aluc*
*luteogularis*	*Alus*
*luteosignifer*	*Alut*
*lynchi*	*Alyn*
*lyra*	*Alyr*
*macilentus*	*Amai*
*macrini*	*Aman*
*macrolepis*	*Amal*
*macrophallus*	*Amap*
*maculigula*	*Amau*
*maculiventris*	*Amac*
*magnaphallus*	*Amag*
*marcanoi*	*Amaa*
*mariarum*	*Amar*
*marmoratus*	*Amam*
*marron*	*Amao*
*marsupialis*	*Amas*
*matudai*	*Amat*
*maynardi*	*Amay*
*medemi*	*Amed*
*megalopithecus*	*Ameg*
*menta*	*Amen*
*meridionalis*	*Amer*
*mestrei*	*Ames*
*microlepidotus*	*Amip*
*microtus*	*Amic*
*milleri*	*Amil*
*mirus*	*Amir*
*monensis*	*Amoe*
*monteverde*	*Amot*
*monticola*	*Amon*
*morazani*	*Amor*
*muralla*	*Amur*
*nasofrontalis*	*Anas*
*naufragus*	*Anau*
*neblininus*	*Anei*
*nebuloides*	*Aneu*
*nebulosus*	*Aneb*
*nelsoni*	*Anel*
*nicefori*	*Anic*
*nitens*	*Anit*
*noblei*	*Anob*
*notopholis*	*Anot*
*nubilis*	*Anub*
*occultus*	*Aocc*
*ocelloscapularis*	*Aoce*
*oculatus*	*Aocu*
*olssoni*	*Aols*
*omiltemanus*	*Aomi*
*onca*	*Aonc*
*opalinus*	*Aopa*
*ophiolepis*	*Aoph*
*oporinus*	*Aopo*
*orcesi*	*Aorc*
*ortoni*	*Aort*
*otongae*	*Aoto*
*pachypus*	*Apac*
*paravertebralis*	*Apaa*
*parilis*	*Apai*
*parvicirculatus*	*Apar*
*paternus*	*Apat*
*pentaprion*	*Apen*
*peraccae*	*Aper*
*petersi*	*Apet*
*philopunctatus*	*Aphi*
*phyllorhinus*	*Aphy*
*pigmaequestris*	*Apig*
*pijolense*	*Apij*
*pinchoti*	*Apin*
*placidus*	*Apla*
*poecilopus*	*Apoe*
*pogus*	*Apog*
*polylepis*	*Apol*
*polyrhachis*	*Apoh*
*poncencis*	*Apon*
*porcatus*	*Apor*
*porcus*	*Apoc*
*princeps*	*Apri*
*proboscis*	*Apro*
*propinquus*	*Aprp*
*pseudokemptoni*	*Apsk*
*pseudopachypus*	*Apsp*
*pseudotigrinus*	*Apse*
*pulchellus*	*Apul*
*pumilus*	*Apum*
*punctatus*	*Apun*
*purpurescens*	*Apur*
*purpurgularis*	*Apug*
*pygmaeus*	*Apyg*
*quadriocellifer*	*Aqud*
*quaggulus*	*Aqua*
*quercorum*	*Aque*
*reconditus*	*Arec*
*rejectus*	*Arej*
*rhombifer*	*Arho*
*richardi*	*Arih*
*ricordi*	*Aric*
*rimarum*	*Arim*
*rivalis*	*Ariv*
*roatanensis*	*Aroa*
*rodriguezi*	*Arod*
*roosevelti*	*Aroo*
*roquet*	*Aroq*
*rubribarbaris*	*Arua*
*rubribarbus*	*Arub*
*ruibali*	*Arul*
*ruizi*	*Arui*
*rupinae*	*Arup*
*sabanus*	*Asab*
*sagrei*	*Asag*
*salvini*	*Asal*
*santamartae*	*Asan*
*schiedi*	*Asch*
*schmidti*	*Ascm*
*schwartzi*	*Ascw*
*scriptus*	*Ascr*
*scypheus*	*Ascy*
*semilineatus*	*Asem*
*sericeus*	*Aser*
*serranoi*	*Asea*
*sheplani*	*Ashe*
*shrevei*	*Ashr*
*simmonsi*	*Asim*
*singularis*	*Asin*
*smallwoodi*	*Asml*
*smaragdinus*	*Asma*
*sminthus*	*Asmi*
*soinii*	*Asoi*
*solitarius*	*Asol*
*spectrum*	*Aspe*
*squamulatus*	*Asqu*
*strahmi*	*Asta*
*stratulus*	*Astr*
*subocularis*	*Asub*
*sulcifrons*	*Asul*
*tandai*	*Atan*
*taylori*	*Atay*
*terraealtae*	*Ater*
*terueli*	*Ateu*
*tetarii*	*Atet*
*tigrinus*	*Atig*
*toldo*	*Atod*
*tolimensis*	*Atol*
*townsendi*	*Atow*
*trachyderma*	*Atrc*
*transversalis*	*Atra*
*trinitatus*	*Atri*
*tropidogaster*	*Atro*
*tropidolepis*	*Atrl*
*tropidonotus*	*Atrp*
*umbrivagus*	*Aumb*
*uniformis*	*Auni*
*unilobatus*	*Aunl*
*utilensis*	*Auti*
*utowanae*	*Auto*
*valencienni*	*Aval*
*vanidicus*	*Avan*
*vanzolinii*	*Avaz*
*vaupesianus*	*Avau*
*ventrimaculatus*	*Aven*
*vermiculatus*	*Aver*
*vescus*	*Aves*
*vicarius*	*Avic*
*villai*	*Avil*
*vittigerus*	*Avit*
*wampuensis*	*Awam*
*wattsi*	*Awat*
*websteri*	*Aweb*
*wellbornae*	*Awel*
*wermuthi*	*Awer*
*whitemani*	*Awhi*
*williamsi*	*Awil*
*williamsmittermeierorum*	*Awim*
*woodi*	*Awoo*
*yoroensis*	*Ayor*
*zeus*	*Azeu*

• Once established, modifications to the four letter abbreviations are strongly discouraged in order to maintain clarity, even in cases of renaming or reclassification.

• This system of nomenclature does not address sub-species designations or geographic 'races.' The AGNC is currently accepting community proposals for these designations.

### Abbreviations for conserved sequences

A subclass of sequences can be defined by their high degree of conservation across taxonomic levels [47,48]. Nomenclature for these conserved sequences (CSs) poses unique challenges because they lack defining content, such as that comprising transposons and repetitive elements. Additionally, CSs are not always completely conserved and occasional duplicate CSs are scattered throughout the genome. We propose to describe CSs in the *Anolis *genome using a combination of species code, unique identification number, length, percent conservation with other species, and characterization of species with which they are shared [49]. We recommend that:

• CS names begin with the species code, *Acar*, to identify *Anolis carolinensis *as the species within which these sequences are described.

• A unique, 1-indexed, arbitrarily assigned number follow the species name.

• Abbreviated length class designations follow the CS number. We define the length classes as follows: (**s**) short ≤ 99 bp; (**m**) medium 100-499 bp; or (**l**) long ≥500 bp).

• A numeral representing percent conservation to the reference species ((1) 100-95%; (2) 94-90%; or (3) 89-85%) follows the length class designation.

• CS names end with an abbreviated indicator of the taxonomic span of conservation: (S) shared among Sauropsida, (M) shared among Mammalia, (B) shared among Batrachia, and (G) shared among Gymnophiona.

Using this nomenclature, the 1,000th CS identified in the *A. carolinensis *genome that is 600 bp long having 100% conservation between *A. carolinensis *and chicken genomes would be named *Acar1000l1SMB*.

### Abbreviations for *Anolis *genetic markers including microsatellite assays

The *A. carolinensis *genome contains many types of repetitive elements including mononucleotide tracts, microsatellites, minisatellites, and satellites. Many researchers focus on simple tandem repeats (STRs, also known as short tandem repeats, microsatellites or simple sequence repeats, SSRs). Some STRs have variable numbers of repeats (i.e., variable number tandem repeats, VNTRs). However, variation is often not reported with the genomic sequence and may be inconsistent among populations and species, and knowledge of variation can change through time as more individuals are sampled. Rather than subdividing and explicitly defining the different repeat types or using VNTR status, we provide a simple, unique nomenclature that can be applied to all STRs in any species of *Anolis*. This nomenclature is linked to a more descriptive, locus-specific annotation available from *lizardbase*. Additional detail regarding the challenges of explicitly defining various classes of STRs has been described [50].

We propose that *Anolis *STRs be assigned a name consisting of three fields separated by underscores:

1) the species code described in Part 4 above derived from the organism of origin,

2) the letters 'str' for simple tandem repeat, and

3) a unique, 1-indexed, identification number

Using this nomenclature, the 8^th ^STR identified in the *A. carolinensis *genome would be coded as *Acar_str_8*. We will store additional, locus-specific information such as repeat unit, genomic location, and number of repeats in a separate database, linked to each STR using these unique names. The submission of STR markers and assignment of unique identification numbers will be handled through *lizardbase *by the AGNC or designated member.

## Conclusions

### Future objectives of the *Anolis *Gene Nomenclature Committee

The recently published green anole (*A. carolinensis*) genome [1] provides an example of how a community of researchers with both common and distinct interests can work together to build an enduring resource. This genomics resource now provides an opportunity for the community to advance a greater knowledge of gene function and orthology. As work progresses on *Anolis *species genomes, new and unforeseen nomenclature issues will certainly arise. The goal of the AGNC is to foster community-based discussion where these problems can be resolved. We have presented guidelines for three immediate objectives for the AGNC but we foresee the need to rapidly address the following objectives:

• Nomenclature for populations and treatment of geographic variation

• Creating a common nomenclature for genetic markers such as microsatellites and SNPs

• Creating a common nomenclature for transposable elements

The AGNC welcomes feedback from the community to raise overlooked issues and unforeseen conflicts. The AGNC views these recommendations as an evolving document, and current, archival, and proposed revisions will be posted to the anole community web sites:

*lizardbase *[28]

*Anolisgenome *[51]

*Anolis Newsletter *[52]

*Anole Annals Blog *[53]

Correspondence to any member of the committee is welcomed. We also would like to elicit comments and suggestions from other research communities with unannotated genomes. It would be helpful to be able to develop and share such important resources and experiences together.

## List of abbreviations used

AGNC: *Anolis *Gene Nomenclature Committee; BAC: bacterial artificial chromosome; ECC: evolutionary character code; CS: conserved sequence; GO: Gene Ontology; HCOP: Human Gene Nomenclature Committee Comparison of Orthology Predictions; HUGO: Human Genome Organization; mya: million years ago; OMA: Orthologs Matrix Project; UCSC: University of California: Santa Cruz; STR: short tandem repeat; VNTR: variable number tandem repeat; ZFIN: Zebrafish Information Network.

## Competing interests

The authors declare that they have no competing interests.

## Authors' contributions

KK, RJK, AA, TCG, DBM, TJS, JW and JWR are members of the *Anolis *Gene Nomenclature Committee and conceived of the report and participated in the drafting of the manuscript. SB, NGC, BCF, DEJ, JL, SP, CJS, and JS have all contributed sections to the report and have participated in the drafting of the manuscript. All authors read and approved the final manuscript.
